# Chronic haloperidol administration downregulates select BDNF transcript and protein levels in the dorsolateral prefrontal cortex of rhesus monkeys

**DOI:** 10.3389/fpsyt.2023.1054506

**Published:** 2023-02-02

**Authors:** Scott E. Hemby, Scot McIntosh

**Affiliations:** Department of Basic Pharmaceutical Sciences, Fred Wilson School of Pharmacy, High Point University, High Point, NC, United States

**Keywords:** neurotrophin (NT), long non-coding RNA, schizophrenia, antipsychotic action, messenger RNA (mRNA), non-human primate (macaque), protein

## Abstract

Post-mortem studies in the prefrontal cortex and hippocampal formation from schizophrenia patients have revealed significant disruptions in the expression molecules associated with cytoarchitecture, synaptic structure, function, and plasticity, known to be regulated in part by brain derived neurotrophic factor (BDNF). Interestingly, several studies using postmortem brain tissue from individuals diagnosed with schizophrenia have revealed a significant reduction in *BDNF* mRNA and protein levels in the dorsolateral prefrontal cortex (DLPFC), hippocampus and related areas; however, differentiating the effects of illness from antipsychotic history has remained difficult. We hypothesized that chronic antipsychotic treatment may contribute to the altered *BDNF* mRNA and protein expression observed in post-mortem brains of individuals diagnosed with schizophrenia. To address the influence of antipsychotic administration on BDNF expression in the primate brain, rhesus monkeys orally administered haloperidol, clozapine, or vehicle twice daily for 180 days. We found *BDNF* splice variants 4 and 5 in the DLPFC and variant 2 in the EC were significantly down-regulated following chronic administration of haloperidol. In addition, proBDNF and mature BDNF expression in the DLPFC, but not the EC, were significantly reduced. Based on the known regulation of BDNF expression by *BDNF-AS*, we assessed the expression of this lncRNA and found expression was significantly upregulated in the DLPFC, but not EC. The results of the present study provide evidence of haloperidol-induced regulation of *BDNF* mRNA and protein expression in the DLFPC and suggest an important role for *BDNF-AS* in this regulation. Given the role of BDNF in synaptic plasticity, neuronal survival and maintenance, aberrant expression induced by haloperidol likely has significant ramifications for neuronal populations and circuits in primate cortex.

## Introduction

Schizophrenia is a chronic, debilitating mental illness consisting of positive symptoms, including psychosis, negative symptoms, and persistent cognitive deficits, such as impairments in working memory. Alterations in circuitry of the dorsolateral prefrontal cortex (DLPFC) and entorhinal cortex as well as changes in the functional connectivity of the prefrontal-hippocampal are thought to contribute to impairments of working memory in individuals diagnosed with schizophrenia (1–4). Abnormalities in parvalbumin positive GABAergic neurons, including reduced expression of *GAD67*, *GAT1*, and parvalbumin mRNA, as well as decreased dendritic branching and reduced somal size in pyramidal neurons, are thought to contribute to prefrontal cortical dysfunction in schizophrenia (5–13). Within the prefrontal-hippocampal circuit, the EC serves as a primary conduit of information flow to the hippocampus (14) and regulates cortical-hippocampal interactions (15). Various deficits have been observed in the EC of individuals diagnosed with schizophrenia, such as increased glutamatergic innervation (16) and cytoarchitectural alterations (3, 4, 17), which have been hypothesized to result from disruption of neuronal migration and differentiation during development (3, 18), along with decreased expression of synaptic and vesicular protein mRNAs (19, 20).

Brain derived neurotrophic factor (BDNF) has been proposed as a potential mechanistic link between reduced expression of GABA neuronal marker mRNAs, altered neuronal architecture and synaptic transmission related proteins at GABA and glutamate synapses with the neuropathology of schizophrenia. Deficits in BDNF have been associated with decreased numbers of GABAergic synapses (21) and GABA neuronal markers including somatostatin, parvalbumin, GAD65, GAD67, and GAT-1 (22, 23), decreased neuronal size and dendritic branching of pyramidal cells (24), as well as decreased vesicular and synaptic protein mRNAs (25, 26). Indeed, previous studies using human postmortem brain tissue from individuals diagnosed with schizophrenia have reported significant decreases in total *BDNF* mRNA (12, 27–29), splice variant transcripts (30, 31) as well as reductions in BDNF protein levels [28, 31, 32; but see (33)] in the DLPFC. Reductions in TrkB mRNA (12, 34) and protein (33) have also been reported in postmortem DLFPC of individuals diagnosed with schizophrenia [see also (27, 30, 35)]. To date, the only published study examining BDNF/TrkB signaling in postmortem EC of individuals diagnosed with schizophrenia reported no change in *BDNF* mRNA but a significant decrease in TrkB mRNA levels in Layer II in this region (35).

Studies utilizing human post-mortem brain samples from individuals diagnosed with schizophrenia have provided significant insight into neuropathological correlates of the disorder; however, discerning whether changes in BDNF expression are due to the pathophysiology of schizophrenia or from chronic antipsychotic administration remains problematic. Various approaches have been used to differentiate disease from drug effects for BDNF. For example, several studies suggest that changes observed in the DLPFC of schizophrenics are not influenced by antipsychotic treatment by referencing a lack of effect of chronic administration of antipsychotics on *BDNF* mRNA expression in the frontal cortex of rodents (33, 36, 37); however, other studies report a reduction in prefrontal BDNF levels (38–42). The conclusion is founded on the assumption of structural and functional equivalency between the primate DLPFC and rodent PFC, an assumption that has been challenged and most recently summarized in a review by Laubach et al. (43). To date, only one published study has examined the effects of chronic antipsychotic administration on *BDNF* mRNA or protein expression in primate brain with the authors reporting no change in *BDNF* mRNA levels in the DLPFC in male cynomolgus monkeys (12). Secondly, in the aforementioned human post-mortem brain studies of schizophrenia, changes in BDNF protein and mRNA expression were attributed to schizophrenia pathophysiology and not to antipsychotic treatment based on (1) the lack of correlation between BDNF expression with last dose, average daily dose and lifetime equivalents of chlorpromazine or fluphenazine (27–29, 31, 35), (2) clinical records and familial report, post-mortem toxicology of blood or brain tissue (31), or the (3) lack of effect of haloperidol administration in rodents (33) and monkeys (12). A lack of correlation between measure of BDNF expression and antipsychotic use prior to death assumes medication adherence. A recent systematic review and meta-analysis of 46 observational studies conducted among adult patients (18 years and older) with major psychiatric disorders revealed psychotropic medication non-adherence rates of 56% for schizophrenia and 44% for bipolar disorder (44), findings consistent with rates reported previously for schizophrenia (45). Due to the significant issue of medication non-adherence in schizophrenia, reliance on medication histories as a surrogate for antipsychotic use is tenuous. In contrast, the use of non-human primates with controlled drug administration histories provides a more reliable means to determine the effects of chronic antipsychotic administration on biochemical measures in a species with brain regions homologous to humans.

A detailed examination of BDNF transcript and protein levels in primate brain has not been explored in a controlled prospective study to date. The lack of such information is surprising given the extensive use of antipsychotics for the treatment of schizophrenia and the inability to conduct such studies in humans. Examining the potential contribution of chronic antipsychotic drug administration on the regulation of BDNF expression is critical for understanding the long-term effects of these drugs in primate brain. The multiple cellular functions produced by BDNF are due in part to the complex, yet integrated, mechanisms regulating transcription and translation. The human *BDNF* gene can generate 17 potential splice variants, each characterized by alternative splicing of 5′ untranslated exon(s) to a 3′ exon encoding the protein and the 3′UTR containing two polyadenylation signals (46). Splice variants are trafficked to distinct cellular compartments for translation – enabling site-specific BDNF-induced neuroadaptations at active dendritic domains (47–49) and providing spatio-temporal regulation of transcription. The *BDNF* gene locus also includes natural antisense transcripts on the opposite strand encoding the long non-coding RNA antisense BDNF (*BDNF-AS*) (46, 50) which provides an additional means of post-transcriptional regulation to decrease *BDNF* mRNA levels by serving as an siRNA and/or *via* chromatin remodeling at critical regulatory regions of the *BDNF* gene (51). Once transcribed, BDNF splice variants are translated into proBDNF and subsequently to mature BDNF, which bind to two distinct receptors and exert opposite effects on synaptic structure and neuronal structure. proBDNF preferentially binds to the p75 pan-neurotrophin receptor (p75^NTR^) and sortilin to decrease dendritic complexity, spine density, and synaptic transmission and facilitate long term depression whereas mature BDNF preferentially binds and activates tropomyosin receptor kinase B (TrkB) promoting neuronal differentiation, survival, synaptic plasticity, and long-term potentiation (52–54).

Current knowledge of the effects of antipsychotics on *BDNF* mRNA and protein expression in primate brain is limited, but critical for understanding the effects of chronic administration in humans. Both first generation antipsychotics (typical), D2 preferential antagonists, and second-generation antipsychotics (atypical), possessing a broader pharmacological profile, are effective in reducing psychotic symptoms and decreasing the occurrence of relapse (55, 56). Chronic administration of typical antipsychotics in rodents adversely affects neuronal plasticity (57–62) and the expression of BDNF in a brain-region specific manner (36, 38, 40–42, 63), whereas atypical antipsychotics exert multiple neuroprotective effects in individuals diagnosed with schizophrenia (64–68), and in both *in vivo* (63, 69–73) and *in vitro* preclinical studies (74–78). Therefore, the present study was undertaken to determine the effect of chronic administration of haloperidol and clozapine, as prototypical first- and second-generation antipsychotics, respectively, on BDNF expression in the DLPFC and EC of rhesus monkeys. We hypothesized that chronic administration of haloperidol, but not clozapine, would selectively decrease *BDNF* splice variant mRNAs, decrease proBDNF and mature BDNF levels and increase *BDNF-AS* RNA in the DLPFC and EC.

## Materials and methods

### Subjects

Rhesus monkeys (*Macaca mulatta*) were housed in individual cages and could hear, see, and touch other monkeys in adjacent cages. Monkeys received food and water *ad libitum*. Monkeys were randomly assigned to one of four treatment groups: drug-naïve control (CTRL, *n* = 10; 6.0 ± 0.5 years), low dose haloperidol (LHAL, *n* = 10; 0.7 mg/kg b.i.d., p.o.; 6.0 ± 0.4 years), high dose haloperidol (HHAL, *n* = 8; 2.0 mg/kg b.i.d., p.o.; 6.2 ± 0.4 years), and clozapine (CLZ, *n* = 10; 2.6 mg/kg b.i.d., p.o.; 6.2 ± 0.4 years) using previously established protocols (79–82). Males and females were equally represented in each group. Antipsychotics were mixed with powdered sugar and given in peanut butter or fruit treats and administered for 6 months. Monkeys in the CTRL group received powdered sugar in peanut butter or fruit treats. Monkeys received social enrichment, human interaction, variety in diet, and age-appropriate objects. Subjects were observed a minimum of twice daily for the duration of the experiments. No overt physical signs of dystonia (abnormal posturing or repetitive movements in the back, neck, jaw, eyes, face, or tongue), drug induced parkinsonism (tremor, rigidity, poverty of movement) or tardive dyskinesias (involuntary choreoathetoid movements) were observed. The care of the animals and euthanasia procedures in this study were performed according to the National Institutes for Health Guide for the Care and Use of Laboratory Animals and were approved by the Institutional Animal Care and Use Committees at Emory University and Wake Forest University.

### Blood and tissue collection

Monkeys were trained to extend a leg and remain stationary during venipuncture through a process of reinforcing successive approximations with food treats. Once trained, blood samples were taken from the lateral saphenous vein and collected in silicone coated serum vacutainer tubes for serum and K3EDTA coated vacutainer tubes (BD Biosciences, Haryana, India), for plasma. Samples were collected prior to the morning dose of the antipsychotic and therefore represented trough levels. Blood in silica coated tubes was centrifuged at 1600 x *g* for 30 min. Serum was collected apportioned in 0.5 ml aliquots then stored at −80°C until assayed. Blood in K_3_EDTA coated tubes was centrifuged 1000–2000 x *g* for 10–15 min at 4°C, then apportioned in 0.5 ml aliquots and transferred to sterile polypropylene tubes. Aliquots used for blood chemistry analysis were stored at 4°C and shipped accordingly while aliquots for analysis of drug concentrations or biochemical assays were stored at −80°C until assayed.

After the 6-month treatment protocol, monkeys were sedated with ketamine followed by an overdose of sodium pentobarbital then transcardially perfused with ice cold phosphate buffered saline. Brains were removed and cut into four mm thick coronal slabs using a brain matrix (Ted Pella, Redding, CA, USA; Cat# 15039). Brain slabs were rapidly frozen on metal plates cooled by dry ice and stored at −80*^o^*C. Time from the beginning of transcardial perfusion to freezing of brain tissue was designated as the post-mortem interval (PMI) and ranged from 38 to 87 min and did not differ significantly between groups. Brain tissue was excised using a Miltex 3.5 mm diameter short handle biopsy punch. Care was taken to maximize consistency of the punch procedure across all brains. The ventral and dorsal banks of the principal sulcus were dissected as representative of DLPFC (Area 46). Brain tissue corresponding to the rostral and intermediate EC (Area 28) was dissected, immediately rostral to the appearance of the hippocampus, located between the rhinal fissure and the amygdala, according to anatomical landmarks and stereotaxic atlas (83).

### Plasma drug levels

Blood samples were collected on the day of necropsy prior to antipsychotic administration. Plasma concentrations of clozapine and norclozapine were determined by gas-liquid chromatography (84, 85). Plasma concentrations for haloperidol and reduced haloperidol were determined by radioimmunoassay as described previously (86, 87). The limits of quantification were 0.3 ng/ml, and intra- and inter-assay CV values were 5.64% at 0.3 ng/ml for both compounds.

### Quantitative real-time PCR (qPCR)

Pulverized tissue from DLPFC and EC from each subject was used for total RNA isolation using Trizol (ThermoFisher, Waltham, MA, USA; Cat# 15596026) followed by chloroform extraction, isopropanol precipitation and ethanol precipitation. RNeasy MinElute Cleanup kits (Qiagen, Cat# 74204) were used to further purify and concentrate RNA. RNA concentration for each sample was determined using a Denovix DS-11 Series Spectrophotometer (Wilmington, DE, USA). For inclusion, samples had 260:280 ratios between 1.9 and 2.2 and 260/230 ratio between 2.0 and 2.2. Two μg of total RNA from each subject as well as RNA pooled from each subject were reverse transcribed with Superscript IV VILO Master Mix with ezDNAse (ThermoFisher, Waltham, MA, USA; Cat# 11766050). cDNA samples for each subject from each region were diluted 1:5–1:10 with RNAse-free water. Serial dilutions of the pooled cDNA were aliquoted for generating standard curves. Reference genes were selected from eleven candidate reference transcripts from rhesus monkeys (Table 1) using the geNorm analysis algorithm from qBase+ (version 3.1, BioGazelle, Ghent, Belgium) which enables the determination of the most stable reference genes from a selected test panel of genes, as described previously (88–91). By computing the average pairwise variation for each control gene paired with all other tested control genes, qBase + calculates the gene expression stability measure *M* allowing for the selection of the most stably expressed control genes in a given sample set, thus minimizing any bias in the data as a result of normalization. The gene expression normalization factor is calculated based on the geometric mean of a user-defined number of reference genes (92). TaqMan assays were used to assess transcript levels of *BDNF* variants (1–6, 9, 12; Table 2). A preliminary screen of these transcripts revealed variants 6, 9, and 12 were not reliably detected in either brain region. Therefore, subsequent analyses focused on *BDNF* variants 1–5. Thermal cycling conditions were as followed: one cycle: 50°C for 30 s, 95°C for 3 s followed by 40 cycles of 2 s at 95°C and 20 s at 60°C. *PSMC4* and *CDKNB* were the most stably expressed transcripts and served as reference genes for the DLPFC, while *PSMC4* and *EIF2B1* served as reference genes for the EC. Each transcript along with the two reference genes from the respective region were assayed in triplicate on a 384 well PCR plate. For each transcript of interest, samples from each experimental group were assayed on the same qPCR plate. On each plate, aliquots of cDNA from each subject along with TaqMan Assays, TaqMan Fast Advanced Master Mix (ThermoFisher, Waltham, MA, USA; Cat# 444963), and DNAse/RNase free water were combined to a final volume of 10 μl and subjected to 95°C for 30 s for one cycle followed by 40 cycles of 95°C for 3 s and 60°C for 20 s with a temperature ramp of 1.6°C/s. Serial dilutions of the pooled cDNA (for standard curves) were run in triplicate along with no template controls. Normalized quantity means for each subject for each transcript were calculated using the relative standard curve method. To exclude measurement errors, the percent variance was determined for the triplicates of each transcript. If one replicate exhibited more than 50% variance, the replicate was removed, and duplicates were used for analysis. If the variance of the duplicates remained greater than 50%, the entire sample was removed from analysis. Data for each gene of interest was expressed as the quantity mean for the gene of interest/geometric mean of quantity mean values for the selected reference genes. In the DLFPC, data were excluded from one subject in the CLZ group for variant 1 and from one subject in the HHAL group for variant 5 based on the exclusion criteria described in the Section “Materials and methods.” In the EC, data were excluded from one subject in the LHAL group for variant 3 based on the exclusion criteria described in the Section “Materials and methods.”

**TABLE 1:** TaqMan assay reference genes.

Gene symbol	Gene name	TaqMan assay
*ABL1*	ABL proto-oncogene 1, non-receptor tyrosine kinase	Rh02856943_m1
*CASC3*	CASC3 exon junction complex subunit	Rh02863431_m1
*CDNK1B*	Cyclin dependent kinase inhibitor 1B	Rh04344806_m1
*EIF2B1*	Eukaryotic translation initiation factor 2B subunit alpha	Rh00426752_m1
*GAPDH*	Glyceraldehyde-3-phosphate dehydrogenase	Rh02621745_g1
*HPRT1*	Hypoxanthine phosphoribosyltransferase 1	Rh02800695_m1
*MRPL19*	Mitochondrial ribosomal protein L19	Rh00608520_m1
*PGK1*	Phosphoglycerate kinase 1	Rh02621707_g1
*POLR2A*	RNA polymerase II subunit A	Rh01108273_m1
*PP1B*	Protein phosphatase 1 catalytic subunit beta	Rh01018503_m1
*PSMC4*	Proteasome 26S subunit, ATPase 4	Rh02793060_m1

**TABLE 2:** Human BDNF variant mRNAs.

Human BDNF variant	Exon(s)	NCBI Ref Seq	TaqMan assay
1	IXabcd	NM_170735.6	Hs00542425_s1
2	IIc-IXd	NM_170732.4	Hs00538278_m1
3	I-IXd	NM_170731.5	Hs00538277_m1
4	VIb-IXd	NM_001709.5	Hs00156058_m1
5	IV-IXd	NM_170733.4	Hs00380947_m1

The five BDNF splice variant mRNAs investigated in the present study. For each variant, the 5′ exon followed by the 3′ exon that compose the respective variants the corresponding NCBI reference sequence accession number and TaqMan assay identifier are provided. Nomenclature according to Pruunsild et al. (46).

Referencing the UCSC Genome Browser (93),^1^ rhesus monkey sequences were determined *in silico* using the human cDNA sequence of interest to query the rhesus monkey genome assembly (February 2019; Mmul_10/rheMac10) using the BLAT search engine (94). Primers for pan *BDNF* and *BDNF-AS* were designed for rhesus monkey sequence using the PrimerQuest Tool and purchased from Integrated DNA Technologies (Coralville, IA, USA). For pan *BDNF*, the following primer pairs were used: 5′-GCTGCAAACATGTCCATGAGGGTC-3′ (forward), 5′-TTGGC CTTTCGATACAGGGACCTTT-3′ (reverse), targeting the coding DNA sequence portion of exon IX that is common to all *BDNF* variant mRNAs. Data from one subject in the CTRL group and one subject in the LHAL were excluded in the analysis for the DLPFC and EC based on the exclusion criteria described above. For *BDNF-AS*, the following primer pairs were used: 5′-AACTGGCTAGAGCAATGTATC-3′ (forward), 5′-TAGGGAGCCAACAAACAAC-3′ (reverse). Data from one subject in the CTRL group was excluded in the analysis for the DLPFC and EC based on the exclusion criteria described above. Primers were designed to anneal to BDNF-AS sequence downstream of the *BDNF* gene (exons 1–4) Splicing of exons 2 and 4 represents a major splicing pattern for the *BDNF-AS* transcript in humans (46). Primers were combined with PowerUp SYBR Green Master Mix (ThermoFisher, Waltham, MA, USA; Cat# A25776), cDNA from each sample and DNAse/RNase free water to a final volume of 10 μl and subjected to one cycle of 50°C for 2 min and 95°C for 2 min followed by 40 cycles of 95°C for 15 s and 60°C for 1 min with a temperature ramp of 1.6°C/s. Melt curves were generated for *BDNF-AS* and controls for SYBR green assays (Supplementary material; Figures 2, 3). All qPCR reactions were conducted on the ABI QuantStudio 6 Flex Real-Time PCR system with a 384 well block (ThermoFisher, Waltham, MA, USA). Fluorescence was measured during the 60°C step for each cycle. Reactions were quantified by the relative standard curve method using QuantStudio software v.1.1 generating a mean quantity value (Qty mean) from the triplicates of each sample for each gene of interest. Data for each gene of interest was expressed as the quantity mean for the gene of interest/geometric mean of quantity mean values for the selected reference genes.

**FIGURE 1:**
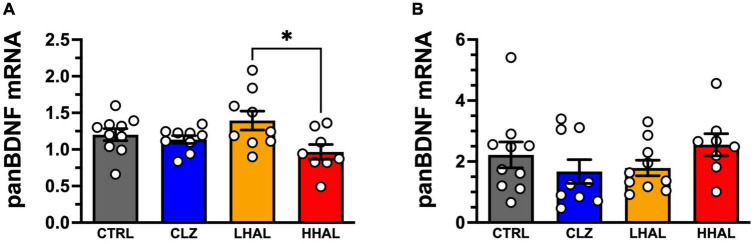
panBDNF mRNA levels in the DLPFC and EC of rhesus monkeys following 6-month antipsychotic administration regimen. Chronic antipsychotic administration resulted in a significant change in pan *BDNF* mRNA expression in the DLPFC [*F*_(3,32)_ = 3.321, *P* = 0.0320] **(A)**. *Post hoc* analysis revealed a significant difference between the groups was LHAL > HHAL (*P* = 0.0197); however, none of the groups were significantly different from CTRL. In the EC, no significant differences in panBDNF expression were detected following chronic antipsychotic administration [*F*_(3,33)_ = 1.163, *P* = 0.3385] in the EC **(B)**. pan *BDNF* expression values are presented as bars which represent the mean (±S.E.M.) values following determination from relative standard curves. For each subject, expression values were calculated as quantitative mean value of pan*BDNF* divided by the average of the quantitative mean values for the two reference genes for the respective regions. Bars represent the mean ± S.E.M. for the respective groups. Circles denote expression levels for individual subjects per group. *p < 0.05.

**FIGURE 2:**
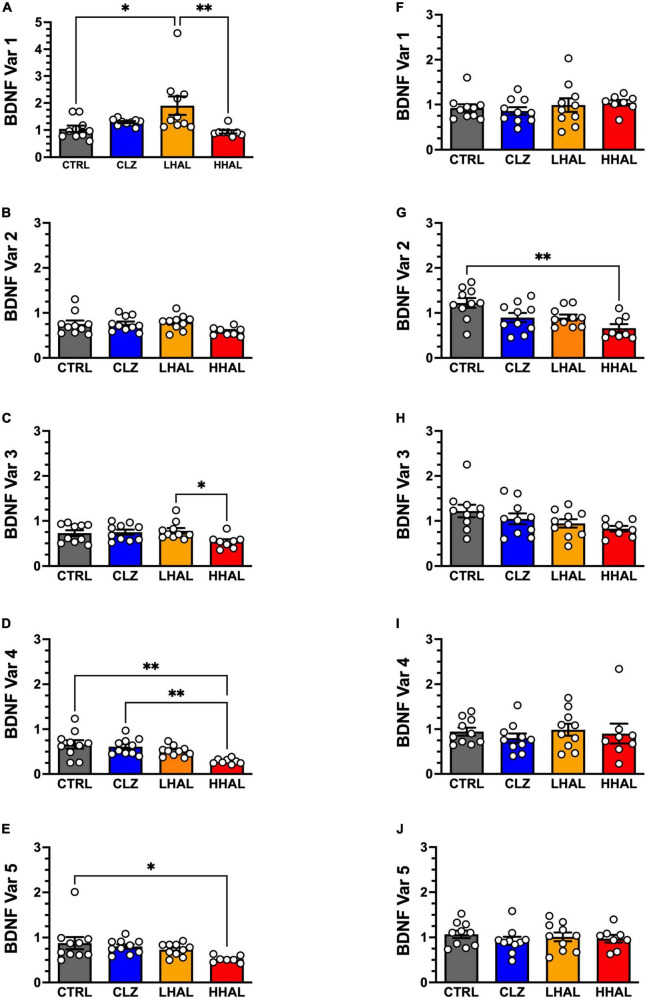
*BDNF* variant mRNA levels in the DLPFC and EC of rhesus monkeys following 6-month antipsychotic administration regimen. *BDNF* variants 1–5 were assessed in the DLPFC and EC using qPCR. In the DLPFC **(A–E)**, significant differences between the groups were identified for *BDNF* variant 1 [*F*_(3,34)_ = 6.88, *P* = 0.0011], variant 4 [*F*_(3,37)_ = 6.635, *P* = 0.0012], and variant 5 [*F*_(3,35)_ = 5.5, *P* = 0.0037]. Tukey’s multiple comparison test revealed the followings differences: Variant 1: LHAL > CTRL (*P* = 0.0163), (*P* = 0.0094); Variant 4: CTRL > HHAL (*P* = 0.0011), CLZ > HHAL (*P* = 0.0052); Variant 5: CTRL > HHAL (*P* = 0.0355). No significant difference among groups were detected for *BDNF* variants 2 and 3 in the DLPFC. For the EC **(F–J)**, a significant difference between the groups was identified for *BDNF* variant 2 [*F*_(3,34)_ = 5.805, *P* = 0.0026]. Tukey’s multiple comparison test revealed CTRL > HHAL (*P* = 0.0014). No significant difference among groups was observed for the other *BDNF* variants in the EC. *BDNF* variant values are presented as bars which represent the mean (±S.E.M.) values following determination from relative standard curves. For each subject, *BDNF* variant expression values were calculated as quantitative mean value of the variant divided by the average of the quantitative mean values for the two reference genes for the respective regions. Bars represent the mean ± S.E.M. for the respective groups. Circles denote expression levels for individual subjects per group. *p < 0.05, ^**^p < 0.01.

**FIGURE 3:**
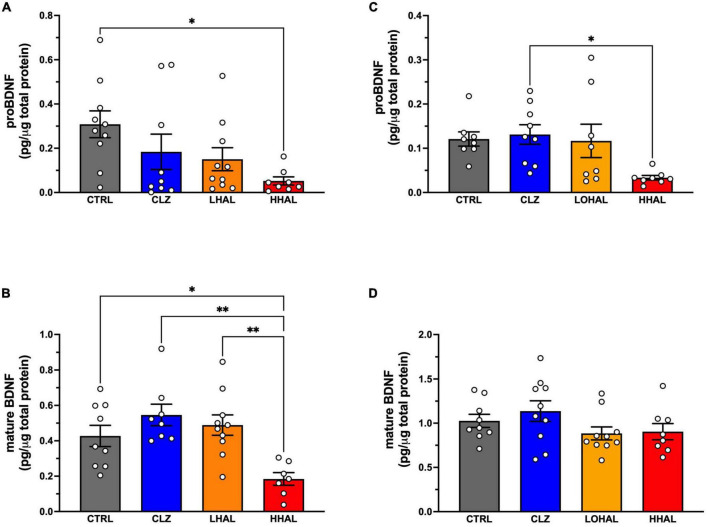
proBDNF and mature BDNF levels are decreased following chronic administration of 4.0 mg/kg/day haloperidol. In the DLPFC, proBDNF **(A)** was significantly different among the groups [*F*_(3,34)_ = 3.631, *P* = 0.0235] and Tukey’s multiple comparison test revealed CTRL > HHAL (*P* = 0.0161). Mature BDNF **(B)** was also significantly different among the groups in this region [*F*_(3,33)_ = 6.889, *P* = 0.0012] where Tukey’s multiple comparison test revealed CTRL > HHAL (*P* = 0.0011) and LHAL > HHAL (*P* = 0.0271). In the EC, proBDNF **(C)** was significantly different among the groups [*F*_(3,29)_ = 3.684, *P* = 0.0231] and Tukey’s multiple comparison test revealed CLZ > HHAL (*P* = 0.0271). No significant difference in mature BDNF **(D)** between the groups was observed following chronic antipsychotic administration [*F*_(3,33)_ = 1.697, *P* = 0.1867]. For each subject, proBDNF and mature BDNF expression levels were calculated as pg/μg tissue protein. Bars represent the mean ± S.E.M. for the respective groups. Circles denote expression levels for individual subjects per group. *p < 0.05, ^**^p < 0.01.

To determine the specificity of the qPCR assay for the amplicons of interest, melt curve analysis was conducted for all primer sets used in the study (95). Separate qPCR reactions were run for each BDNF variant and endogenous control TaqMan assays. Each TaqMan assay was combined with PowerUp SYBR Green Master Mix (ThermoFisher, Waltham, MA, USA; Cat# A25776), cDNA from individual subjects and DNAse/RNase free water to a final volume of 10 μl and subjected to one cycle of 50°C for 2 min and 95°C for 2 min followed by 40 cycles of 95°C for 15 s and 60°C for 1 min with a temperature ramp of 1.6°C/s. For pan *BDNF* and *BDNF-AS*, a melt curve analysis set was included in the qPCR method. For all primer sets, melt curve analysis began immediately following the last extension step. The thermal cycler program consisted of three steps as follows: (1) 1.6°C/s ramp from last extension step to 95°C for 15 s, (2) 1.6°C/sec ramp to 60°C for 60 s, and (3) 0.05°C/s ramp to 95°C for 15 s and fluorescence was continuously acquired. Following completion of the melt curve stage, the negative first derivative of the change in fluorescence as a function of temperature was plotted to produce the melt curve for each primer set for each sample. When two or more peaks were observed in a melt curve analysis, qPCR product was electrophoresed (3V/cm) on a 3% agarose gel with SYBR™ Safe DNA gel stain (Thermofisher, Waltham, MA, USA; Cat#S33102) in 1X TBE buffer (90 mM Tris base, 90 mM boric acid, Na_2_EDTA; pH = 8.3) to determine the presence and size of one or more amplicons. Gels were imaged using the ChemiDoc XRS + Imager (BioRad, Hercules, CA, USA; Cat#1708265).

### Protein expression

#### Serum prolactin

Antipsychotic-induced hyperprolactinemia is a proxy indicator of D_2_ receptor occupancy manifesting when D_2_ receptor occupancy is greater than 72% (96). Serum prolactin was assessed as a surrogate for central nervous system D2 receptor binding affinity for haloperidol and clozapine. Prolactin levels were determined from serum samples using the MILLIPLEX MAP Human Circulating Cancer Biomarker Magnetic Bead Panel 1 (EMD Millipore, Burlington, MA, USA; Cat# HCCBP1MAG58K) containing prolactin coupled with the Luminex LX200 (EMD Millipore, Burlington, MA, USA) platform in a magnetic bead format according to manufacturer’s instructions. All serum samples were assayed in duplicate, and a pooled serum sample was included as a positive control. Concentrations are expressed as ng/ml. Data were analyzed using xPONENT v.3.1 software (EMD Millipore, Burlington, MA, USA). The minimum detectable concentration for prolactin was 30.2 pg/ml. The coefficient of determination for prolactin was 0.9999. The intra-assay precision was 4.9–15% and the inter-assay precision 4.1–16.2%. Intra-assay precision is generated from the mean of the %CV’s from 16 reportable results across two different concentrations of analytes in a single assay. Inter-assay precision is generated from the mean of the %CV’s across two different concentrations of analytes across 10 different assays.

#### proBNDF and mature BDNF ELISA

Total protein was isolated from pulverized tissue of the DLPFC and EC from each subject. Mature BDNF and proBDNF were assayed using the BiosSensis Mature BDNF/proBDNF Combo *Rapid™* ELISA Kit (Biosensis, Thebarton, SA, Australia; BEK-2441, RRID:AB_2923547). For detection of mature BDNF, total BDNF was acid extracted as previously described (97). proBDNF was extracted using RIPA buffer (50 mM Tris-HCl, 150 mM NaCl; 1.0% NP- 40; 0.5% sodium deoxycholate; pH 7.5 to 8.0). Protease and phosphatase inhibitors (ThermoFisher, Waltham, MA, USA; Cat# 78442) were added to the extraction buffers. Samples were sonicated twice for 15–20 s and incubated on ice for 30 s between sonication. Samples were then centrifuged at 15,000 rpm for 10 min at 4°C and the supernatant (total protein lysate) was transferred to a new tube. Protein concentrations were measured using the bicinchoninic acid protein assay kit (ThermoFisher, Waltham, MA, USA; Cat# 23275) on a spectrophotometer (iD5, Molecular Devices, Sunnyvale, CA, USA). Aliquots of 100 μl of isolated protein from each region were transferred to a 96-well ELISA plates. Final absorbances were read at 450 nm using a spectrophotometer (iD5, Molecular Devices, Sunnyvale, CA, USA). The abundance of proBDNF and mature BDNF were normalized to the amount of total protein.

### Statistical analysis

Experiments determining relative gene expression for each BDNF splice variant in each region were run independently. A two-way analysis of variance (Sex × Group) was used to determine differences in mRNA and protein levels in each region. A one-way analysis of variance comparing group means for CTRL, CLZ, LHAL, and HHAL was performed to determine differences in expression levels for splice variants in each region and for serum prolactin levels. Tukey’s multiple comparison test was used for *post hoc* comparisons. Experiments determining levels of proBDNF and mature BDNF in each region were run independently using a one-way analysis of variance comparing group means for CTRL, CLZ, LHAL, and HHAL. Tukey’s multiple comparison test was used for *post hoc* comparisons. A two tailed *t*-test was used to assess differences in *BSNF-AS* expression for each region. Null hypotheses were rejected if *p* < 0.05. All statistical analyses were performed using Prism 9.3.1 for macOS.

## Results

To test the hypothesis that antipsychotic administration altered *BDNF* mRNA and protein expression in primate DLPFC and EC, we utilized cohorts of rhesus monkeys. All groups consisted of equal numbers of female and male rhesus monkeys. The groups did not differ by age [*F*_(3,34)_ = 0.060, *P* = 0.981], weight prior to first administration [*F*_(3,34)_ = 0.0598, *p* = 0.981], weight at necropsy [*F*_(3,34)_ = 0.878, *P* = 0.462] or change in weight from the beginning to the end of the study [*F*_(3,34)_ = 0.8613, *P* = 0.8613].

### Plasma levels of haloperidol and clozapine

In the HHAL group, plasma steady state trough levels were 1.8 ± 0.3 ng/ml and for reduced haloperidol 0.6 ± 0.1 ng/ml. Neither haloperidol nor reduced haloperidol were detectable in the LHAL group. Trough levels as low as 0.86 ng/ml have been reported to be therapeutically effective in patients with schizophrenia (98, 99) and similar to levels reported previously following chronic administration in monkeys (100). No significant differences in serum levels of haloperidol (*t* = 0.5681, df = 6, *P* = 0.5906) or reduced haloperidol (*t* = 0.1325, df = 6, *P* = 0.899) between the sexes was found. In the CLZ group, plasma steady state trough levels of clozapine and norclozapine were 47.8 ± 12.5 ng/ml and 107.0 ± 16.2 ng/ml, respectively. Steady state clozapine plasma trough levels of 50 ng/ml represent the lower end of therapeutically effective levels defined as preventing psychosis relapse (101), although clinical response in patients does not display a strong correlation with clozapine dose. Studies suggest that levels of approximately 350 ng/ml increase the probability of a stable antipsychotic effect (102). No significant sex differences in plasma levels of clozapine (*t* = 2.014, *df* = 3, *P* = 0.1374) or norclozapine (*t* = 0.5987, *df* = 3, *P* = 0.5916) were detected.

### Serum prolactin levels

One-way ANOVA revealed a statistically significant difference in serum prolactin levels between the groups [*F*_(3,20)_ = 3.673, *P* = 0.0295]. Tukey’s multiple comparison test revealed no statistically significant differences between groups; however, serum prolactin in the HHAL group trended toward higher levels vs. CTRL (*P* = 0.0547) and CLZ (*P* = 0.0613) and to a lesser extent LHAL (*P* = 0.1045).

### BDNF gene expression

Exon composition of the human *BDNF* variants is based on the nomenclature reported previously (46; Table 2). *BDNF* variant 1 (exon IXabcd), variant 2 (exons IIc, IXd), variant 3 (exons I, IXd) variant 4 (exons VIb, IXd), and variant 5 (exons IV, IXd) were reliably detected in both the DLPFC and EC. Amplification efficiencies of all primer sets used in this study were between 95.18 and 108.44% (Supplementary material; Table 1). Melt curve analysis plots of primer pairs showed the presence of only one peak indicating a single amplicon was generated (Supplementary material; Figure 1), with the exception of BDNF variant 4 for which two peaks were present (Supplementary material; Figure 2A). Electrophoresis of BDNF variant 4 qPCR product indicated the presence of only one band (Supplementary material; Figure 2B) suggesting the dual peaks observed in the melt curve analysis were not due to the presence of two amplicons, but instead is due to melting of the cDNA in multiple phases likely caused by the presence of G/C rich regions, amplicon misalignment in A/T rich regions and/or secondary structure in the amplicon (95, 103).

mRNA from pan *BDNF* and *BDNF* variants 1–5 were reliably detected in both the DLPFC and EC. In the DLPFC, there were no significant differences between the groups by sex for pan *BDN*F [*F*_(3,28)_ = 1.475, *P* = 0.2427] or *BDNF* variant expression (Var1: [*F*_(3,29)_ = 0.6825, *P* = 0.5699], Var2: [*F*_(3,30)_ = 0.9938, *P* = 0.4365], Var3: [*F*_(3,30)_ = 0.04564, *P* = 0.9868], Var4: [*F*_(3,30)_ = 0.7924, *P* = 0.5077] and Var5: [*F*_(3,29)_ = 1.317, *P* = 0.2877]). Therefore, the data for males and females for each group was combined. In the EC, no significant differences between the groups by sex were observed for pan*BDNF* [*F*_(3,29)_ = 1.503, *P* = 0.2346] or *BDNF* variant expression (Var1: [*F*_(3,30)_ = 0.6258, *P* = 0.6039], Var2: [*F*_(3,30)_ = 0.5285, *P* = 0.6662], Var3: [*F*_(3,30)_ = 0.5605, *P* = 0.6452], Var4: [*F*_(3,30)_ = 1.481, *P* = 0.2397] and Var5: [*F*_(3,30)_ = 1.813, *P* = 0.1662]). Therefore, data for males and females for each group were combined.

pan *BDNF* mRNA expression in the DLPFC was significantly altered by chromic antipsychotic administration [*F*_(3,32)_ = 3.321, *P* = 0.0320]. *Post hoc* analysis revealed a significant difference between the groups was LHAL > HHAL (*P* = 0.0197); however, none of the groups were significantly different from CTRL (Figure 1A). In contrast, no significant differences were detected between the groups for panBDNF [*F*_(3,33)_ = 1.163, *P* = 0.3385] in the EC (Figure 1B).

In the DLPFC, antipsychotic administration significantly altered expression of *BDNF* variant 1 [*F*_(3,34)_ = 6.88, *P* = 0.0011], variant 3 [*F*_(3,34)_ = 2.914, *P* = 0.0483], variant 4 [*F*_(3,37)_ = 6.635, *P* = 0.0012], and variant 5 [*F*_(3,35)_ = 5.5, *P* = 0.0037] (Figures 2A–E). No significant difference was detected for variant 2 [*F*_(3,34)_ = 2.22, *P* = 0.1039]. *Post hoc* analysis revealed the following differences between groups: Variant 1: LHAL > CTRL (*P* = 0.0163), LHAL > HHAL (*P* = 0.0094); Variant 4: CTRL > HHAL (P = 0.0011), CLZ > HHAL (*P* = 0.0052); Variant 5: CTRL > HHAL (*P* = 0.0355). No significant differences were detected between groups for Variant 3. In the EC, there was a significant effect of antipsychotic administration on the expression of variant 2 [*F*_(3,34)_ = 5.805, *P* = 0.0026]. Tukey’s multiple comparison test revealed variant 2 expression levels in HHAL were significantly reduced relative to CTRL (*P* = 0.0014). No significant differences were detected for variant 1 [*F*_(3,34)_ = 0.5135, *P* = 0.6638], variant 3 [*F*_(3,34)_ = 2.135, *P* = 0.1140], variant 4 [*F*_(3,34)_ = 0.3610, *P* = 0.7815], or variant 5 [*F*_(3,34)_ = 0.4767, *P* = 0.7006] (Figures 2F–J).

### BDNF protein expression

ProBDNF and mature BDNF were detected in the DLPFC and EC in the CTRL and HHAL groups. No significant differences between the groups by sex were observed in the DLPFC (proBDNF: [*F*_(3,29)_ = 0.5688, *P* = 0.6400]; mature BDNF: [*F*_(3,27)_ = 0.4805, *P* = 0.6985]) or EC (proBDNF: [*F*_(3,25)_ = 0.1141, *P* = 0.9510]; mature BDNF: [*F*_(3,29)_ = 2.036, *P* = 0.1307]). Therefore, data for males and females for each group were combined. In the DLPFC, proBDNF expression was significantly different among the groups [*F*_(3,33)_ = 3.169, *P* = 0.0371] with *post hoc* analysis revealing expression was significantly lower in the HHAL group compared to CTRL (*P* = 0.0161) (Figure 3A). Similarly, mature BDNF expression in the DLFPC as significantly altered following antipsychotic administration [*F*_(3,31)_ = 6.790, *P* = 0.0012] and *post hoc* analysis revealed expression in the HHAL groups was significantly lower than CTRL (*P* = 0.036) as well as CLZ (*P* = 0.0011) and LHAL (*P* = 0.0154) (Figure 3B). In the EC, antipsychotic administration induced significant alterations in proBDNF expression [*F*_(3,29)_ = 3.684, *P* = 0.0231]; however, the only difference among the groups was a significant decrease in the HHAL group compared to CLZ (*P* = 0.0271) (Figure 3C). No significant difference in mature BDNF expression among the groups was observed following chronic antipsychotic administration [*F*_(3,33)_ = 1.697, *P* = 0.1867] (Figure 3D).

### BDNF-AS lncRNA expression

**FIGURE 4:**
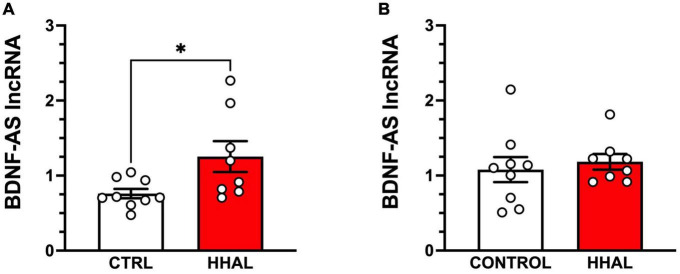
BDNF-AS levels are increased in the DLPFC but not the EC following chronic administration in the HHAL group. In the DLPFC **(A)**, BDNF-AS lncRNA levels were significantly greater in the HHAL group compared to the CTRL group (*t* = 2.414, *df* = 15, *P* = 0.029); however, in the EC **(B)** no significant differences were observed between the HHAL and CTRL groups (*t* = 0.5113, *df* = 15, *P* = 0.6166). For each subject, BDNF-AS levels were calculated as quantitative mean value divided by the average of the quantitative mean values for the two reference genes for the respective regions. Bars represent the mean ± S.E.M. for the respective groups. Circles denote expression levels for individual subjects per group. *p < 0.05.

*BDNF-AS* lncRNA was reliably detected in the DLPFC and EC of all subjects in the CTRL and HHAL groups. No significant differences between the groups by sex were observed in the DLPFC [*F*_(1,13)_ = 1.562, *P* = 0.2334] or EC [*F*_(1,13)_ = 1.125, *P* = 0.3083]. In the DLPFC, *BDNF-AS* was significantly increased in the HHAL group in the DLPFC [*t* = 2.414, *df* = 15, *P* = 0.029] (Figure 4A). No significant changes were observed in the EC [*t* = 0.5113, *df* = 15, *P* = 0.6166] compared to CTRL (Figure 4B).

## Discussion

In this non-human primate study, chronic haloperidol exposure was associated with significant decrease in *BDNF* splice variant mRNA and protein levels in the DLPFC and EC. In the DLPFC, *BDNF* splice variants 4 and 5 as well as proBDNF and mature BDNF protein levels were significantly decreased following chronic haloperidol administration. Interestingly in the EC, *BDNF* variant 2 and proBDNF protein levels were significantly reduced following chronic haloperidol administration; however, no significant changes were observed in mature BDNF. The reductions in *BDNF* mRNA and protein in the DLPFC were inversely correlated with significant elevations in BDNF-AS expression suggesting a potential mechanistic link by which BDNF expression is regulated. Changes were highly specific as changes in DLPFC not observed in the EC and vice versa. Furthermore, clozapine, in contrast to haloperidol, did not significantly alter *BDNF* mRNA or protein expression in either brain region and only the higher dose of haloperidol elicited changes in expression.

Previous studies using human postmortem brain tissue from individuals diagnosed with schizophrenia have reported significant decreases in DLPFC pan-BDNF mRNA (12, 27, 28); however, no changes were reported for in the EC (35). Studies assessing the effect of chronic antipsychotic administration on pan-BDNF mRNA in rodents are equivocal. Chronic administration of haloperidol, but not clozapine, resulted in significant decreases in pan-BDNF mRNA in the CA1, dentate gyrus and CA3 regions of the hippocampus (36, 104, 105), but no change in the medial prefrontal cortex of rats chronically administered haloperidol (36). In contrast, another study reported chronic haloperidol administration in rats did not affect pan-BDNF mRNA expression in the hippocampus but resulted in a significant decreased in expression in the medial prefrontal cortex (40). Given the absence of DLPFC analogue in rodents, assessment of the effects of chronic antipsychotic administration in non-human primates provides a more appropriate determination of the contribution of antipsychotics to the reduced pan-BDNF in humans. A previous study in four male cynomolgus monkeys revealed that chronic haloperidol administration did not alter pan-BDNF mRNA expression in the DLPFC (12). Our results confirm and extend these findings by demonstrating that pan-BDNF mRNA expression was not altered in the DLPFC or EC following chronic haloperidol or clozapine administration in male or female rhesus monkeys. The lack of effect in monkeys following chronic haloperidol administration suggests that the well-documented reductions in pan-BDNF mRNA in humans diagnosed with schizophrenia are due to the illness and not to antipsychotic treatment.

Pan-BDNF expression reflects the summated expression of all BDNF variants and likely does not reflect alterations in discrete BDNF splice variant mRNA expression. Transcription of the human *BDNF* gene involves the splicing of one or more of the ten 5′UTR exons to a 3′ exon IX containing the common coding DNA sequence (CDS) and two different polyadenylation sites (long or short 3′UTRs) yielding 17 variants with either long or shorts UTRs (46). While the CDS encodes for constitutive dendritic targeting of the variants, the respective 5′UTR exons modify the targeting by segregating the variants to predominantly soma, proximal or distal dendritic sites (106). Based on the “spatial code” hypothesis of *BDNF*, stimuli that modify the expression of select variants would enable structural and functional modifications of specific compartments of the dendritic tree (47, 107). Two studies have evaluated the expression of select BDNF variants in brain regions from individuals diagnosed with schizophrenia. In the first study, the expression of BDNF transcripts containing the 5′ exons I, II, IV and VI (corresponding to BDNF splice variants 3, 2, 5, and 4, respectively) in the DLPFC were compared between individuals diagnosed with schizophrenia and controls (31). In separate cohorts of subjects with schizophrenia, the *BDNF* transcript containing the 5′ exon II (variant 2) was significantly reduced in the DLPFC of individuals diagnosed with schizophrenia. The changes in BDNF transcript expression were attributed to schizophrenia based on the lack of correlation with antipsychotic use prior to death. In a separate study, the expression of BDNF transcripts containing the 5′ exons I, IIc, IV, and VI (corresponding to BDNF splice variants 3, 2, 5, and 4, respectively) were assessed in the DLFPC, hippocampus and striatum of individuals diagnosed with bipolar disorder, major depressive illness and schizophrenia and controls (30). In the DLFPC, BDNF transcripts IIc and VI were significantly reduced in individuals diagnosed with schizophrenia. We concur with the conclusion that the changes in *BDNF* variant 2 are likely reflective of the pathology of the illness rather that medication effects since *BDNF* variant 2 expression in the DLPFC was not significantly altered by chronic administration of clozapine or either dose of haloperidol in the present study. In contrast, the decrease in *BDNF* transcript VI (Variant 4) in the DLPFC of individuals diagnosed with schizophrenia as well as monkeys administered haloperidol chronically indicates the altered expression is due to the effects of typical antipsychotics alone or in addition to the pathology of the illness. Determining the relative contribution of disease pathology vs. antipsychotic pharmacology to such remains difficult due to the paucity of availability of post-mortem tissue from drug-naïve individuals diagnosed with schizophrenia as well as the lack of viable non-human primate models of schizophrenia. Lastly, *BDNF* variant 5 (exon IV) was not altered in the DLFPC of individuals diagnosed with schizophrenia but was significantly reduced in the DLFPC of rhesus monkeys following chronic haloperidol administration. We are unable to determine whether the decrease in BDNF variant 2 in EC of monkeys administered haloperidol may also occur as results of disease pathology due to the lack of published reports on BDNF variant expression in this region in individuals diagnosed with schizophrenia.

Given the role of BDNF in neuronal survival, differentiation, and synaptic plasticity (54, 108), the antipsychotic induced reductions in *BDNF* transcript expression in specific brain regions likely has ramifications for neuronal populations that rely on adequate levels of BDNF to maintain their functional and structural integrity. BDNF has been detected in pyramidal neurons of primate cortex with highest expression in Layers III, V, and VI and non-detectable in non-pyramidal neurons (28, 109). A detailed cellular assessment of BDNF variant expression in primate DLFPC and EC has not been reported to our knowledge. Antipsychotic induced reductions in the expression of specific BDNF variants, as reported in the present study, are likely to significantly influence GABAergic and glutamatergic neurons in the DLPFC and EC. For example, variant 5 has been shown to be preferentially located in proximal dendrites of cortical neurons which receive glutamatergic input from local collaterals and adjacent cortical areas. Variant 4 (homologous to rodent *BDNF* exon VII) is preferentially located in the soma (47, 106), which receive input from GABAergic interneurons. Variant 2 is primarily localized to distal dendrites of cortical neurons, which receive both GABAergic and glutamatergic input, where it regulates distal dendritic morphology (47, 106). Determining the identities and characteristics of DLPFC and EC neuronal populations in which changes in BDNF expression occur as well as the temporal profile of those changes are critical for understanding the impact on intracortical and intercortical communication in the prefrontal hippocampal circuit.

Brain derived neurotrophic factor splice variants are translated into proBNDF and subsequently to mature BDNF proteins which bind to their respective receptors and exert opposite effects on synaptic structure and neuronal structure. The results of studies assessing BDNF protein expression in the DLPFC of individuals diagnosed with schizophrenia have been inconsistent. Durany et al. reported significant elevations in BDNF protein in the DLFPC, as well as parietal, temporal, and occipital cortex as well as hippocampus in individuals diagnosed with schizophrenia (32). Separate studies found no change in BDNF protein levels in the DLPFC (33, 110), but a significant decrease in the anterior cingulate cortex and hippocampus (33). All three aforementioned studies utilized ELISA assays and all three failed to indicate the isoform or isoforms that were detected. In contrast, Weickert et al., reported a significant decrease in mature BDNF levels (28) and later reported significant decrease in mature BDNF as well as proBDNF levels (31) in DLFPC of patients with schizophrenia, effects attributed to the disease pathology of schizophrenia. The present findings of reduced proBDNF and mature BDNF in the DLPFC of monkeys administered haloperidol expand this interpretation and indicate the changes are likely due to the effects of typical antipsychotics alone or in addition to the pathology associated with schizophrenia. Furthermore, we postulate that reductions in *BDNF* variant levels may be a significant catalyst for the observed reductions in mature BDNF and proBDNF in the DLPFC. proBDNF preferentially binds to the p75^NTR^ receptor to decrease dendritic complexity, spine density, and synaptic transmission and facilitate long term depression whereas mature BDNF preferentially binds to the TrkB receptor to promote neuronal differentiation, survival, synaptic plasticity, and long-term potentiation (52–54). Given the opposing effects of proBDNF and mature BDNF signaling, the impact of the observed concurrent changes in both isoforms on the structural and functional integrity of the DLPFC is uncertain. Determining the temporal profile of changes in both isoforms as well as the influence of relative ratio of isoform levels would provide insight into how chronic haloperidol impacts BDNF signaling is this region. As noted for changes in BDNF transcript expression in the EC, we are unable to determine whether disease pathology may contribute to the decrease in mature BDNF protein levels due to the lack of published reports on BDNF variant expression in this region in individuals diagnosed with schizophrenia. Future studies assessing BDNF variant and BDNF protein expression in this region are warranted.

The present results are the first demonstration of an association of reduced BDNF transcript and BDNF protein expression and a concomitant increase in the long non-coding RNA *BDNF-AS* in the DLPFC, suggesting a potential role for BDNF-AS in haloperidol induced decreases in BDNF expression in this region. Furthermore, the change in BDNF-AS expression appears to be regionally specific, as similar changes were not observed in the EC despite significant reductions in *BDNF* splice variant 2 and mature BDNF. BD*NF-AS* is located on the antisense strand of *BDNF*, contains 10 exons and can produce hundreds of *cis* natural antisense transcripts, with the majority of the *BDNF-AS* splice variants are complementary to *BDNF* exon IX (46, 50). *BDNF-AS* expression provides an additional level of BDNF transcriptional and translational regulation (46, 51). Although the precise mechanism by which haloperidol-induced *BDNF-AS* expression may contribute to decreased *BDNF* transcription and translation in the present study remains unknown, previous studies have demonstrated *BDNF-AS* can form a duplex with *BDNF* mRNA *in vivo* to knockdown mRNA expression (46) and can recruit EZH2, a histone lysine methyltransferase responsible for the addition of the repressive mark H3K27me3 at the *BDNF* promoter region, thereby repressing transcription (51). Further studies are warranted to determine the expression of BDNF-AS in brain regions of individuals diagnosed with schizophrenia, whether BDNF-AS splice variants directly influence the expression of particular BDNF splice variants or sets of splice variants and to determine the temporal profile of BDNF-AS induction and BDNF splice variant expression as a function of chronic haloperidol administration.

Despite the novelty and potential relevance of the results from this study, there are several issues that may limit interpretation or the extrapolation to the human condition. This study does not take into account the impact of disease state on antipsychotic-induced changes in BDNF expression. Simple summation or subtraction of antipsychotic related effects in rhesus monkeys from determinations made in human postmortem tissue and drug treated animals likely oversimplifies the complexity of interactions between disease state and drug treatment. However, the lack of non-human primate models that recapitulate the etiology, developmental trajectory and manifestation of symptoms as well as the lack of available postmortem brain tissue from first-episode psychosis non-medicated individuals diagnosed with schizophrenia limits the ability to completely differentiate effects that are disease vs. treatment related. In the present study, the effects of antipsychotic administration were evaluated at a single time point and therefore, we cannot be certain that the observed changes in BDNF expression reached or are maintained at a steady state level. Two doses of haloperidol were assessed providing a limited assurance of dose dependent effects; however, the effects observed following clozapine may be limited to the dose evaluated and should be interpreted with caution. Reported changes in *BDNF* expression are confounded by reduced environmental stimulation and social interactions experienced by the rhesus monkeys. Several studies have demonstrated stress-induced alterations in *BDNF* expression in various brain regions in rodents (111–116). Moreover, *BDNF* mRNA and protein levels in several brain areas are increased following environmental enrichment (117–119). The effects of antipsychotic administration were evaluated at a single time point and therefore, we cannot be certain that the observed changes in BDNF expression reached or are maintained at a steady state level.

In conclusion, the present study provides evidence that chronic haloperidol administration induced significant reductions in *BDNF* splice variant mRNAs and BDNF protein levels in the DLPFC and EC of rhesus monkeys. These results are antipsychotic specific, as clozapine did not significantly alter mRNA or protein expression in either region, and dependent on the dose of haloperidol administered chronically, as only the high dose of haloperidol induced the observed changes. Furthermore, *BDNF* splice variant expression BDNF protein isoform expression and *BDNF-AS* expression differed between the two brain regions, suggesting region-specific regulation of BDNF expression. The data suggest that increased expression of *BDNF-AS* may contribute to the reduced expression of BDNF variants 4 and 5 in the DLPFC which contribute to the reduction in proBDNF and mature BDNF protein levels. In the EC, we suggest that reduced expression of BDNF variant 2 expression is associated with decreased mature BDNF levels; however, *BDNF-AS* expression does not appear to contribute to significantly contribute to this reduction in the EC. Speculation on the functional consequences of haloperidol-induced dysregulation of BDNF expression in the DLPFC is premature at this time. Future studies focused on potential changes in BDNF cognate TrkB and p75^NTR^ receptors and their respective intracellular signaling cascades are needed to provide a thorough understanding of antipsychotic induced dysregulation of BDNF signaling in the DLFPC.

## Data availability statement

The raw data supporting the conclusions of this article will be made available by the authors, without undue reservation.

## Ethics statement

The animal study was reviewed and approved by Emory University Institutional Animal Care and Use Committee and Wake Forest University Institutional Animal Care and Use Committee.

## Author contributions

SH was responsible for the study concept and design, selected the TaqMan assays, designed the primers for *BDNF-AS* and pan *BDNF*, performed the data analysis, interpreted the results, and drafted the manuscript. SM provided critical revision of the manuscript for important intellectual content. Both authors contributed to the acquisition of animal data, qPCR, and protein expression data, critically reviewed the content of the manuscript, and approved the final version for submission.

## Footnotes

1
http://genome.ucsc.edu/
