# Supplementary material for: Chronic haloperidol administration downregulates select BDNF transcript and protein levels in the dorsolateral prefrontal cortex of rhesus monkeys
Source: Front Psychiatry. 2023 Feb 2;14:1054506. doi: 10.3389/fpsyt.2023.1054506 (PMC9932326; doi:10.3389/fpsyt.2023.1054506)
Supplement: Supplementary file 1 [file Data_Sheet_1.docx]

**Supplementary Data**

**Table 1. Amplification efficiency of all primer sets used in the study.**

| **TARGET** | **AMP EFF (%)** | **SLOPE** | **R^2^** |
| --- | --- | --- | --- |
| BDNF VARIANT 1 | 95.33 ± 0.10 | -3.349 | 0.990 |
| BDNF VARIANT 2 | 95.18 ± 0.08 | -3.443 | 0.994 |
| BDNF VARIANT 3 | 95.05 ± 0.06 | -3.447 | 0.996 |
| BDNF VARIANT 4 | 96.89 ± 0.09 | -3.399 | 0.993 |
| BDNF VARIANT 5 | 96.20 ± 0.03 | -3.417 | 0.999 |
| panBDNF | 108.44 ± 0.04 | -3.135 | 0.999 |
| BDNF-AS | 100.30 ± 0.09 | -3.315 | 0.994 |
| CDNK1B | 95.68 ± 0.05 | -3.255 | 0.997 |
| EIF2B1 | 102.88 ± 0.04 | -3.255 | 0.997 |
| PSMC4 | 102.85 ± 0.06 | -3.430 | 0.995 |

**Figure 1: Melt Curve Analyses for BDNF Variants 1-3 and 5, panBDNF, BDNF-AS and endogenous controls.** Methods are described in the manuscript.

BDNF Variant 1


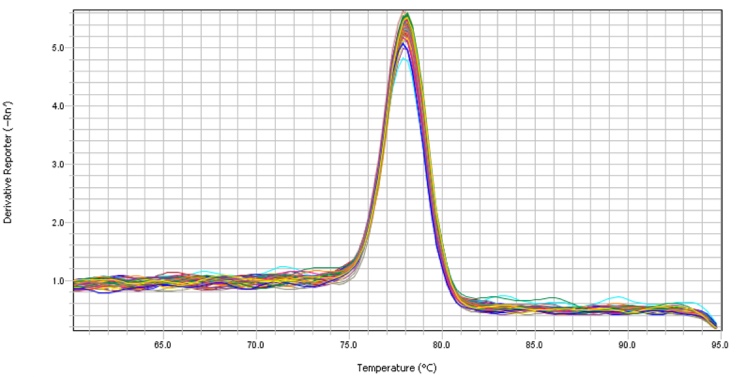


BDNF Variant 2


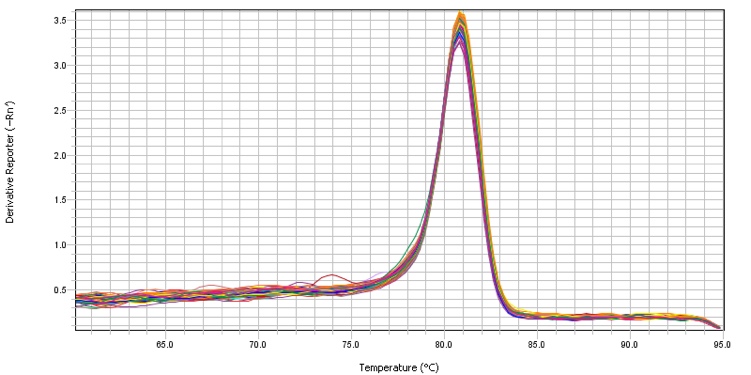


BDNF Variant 3


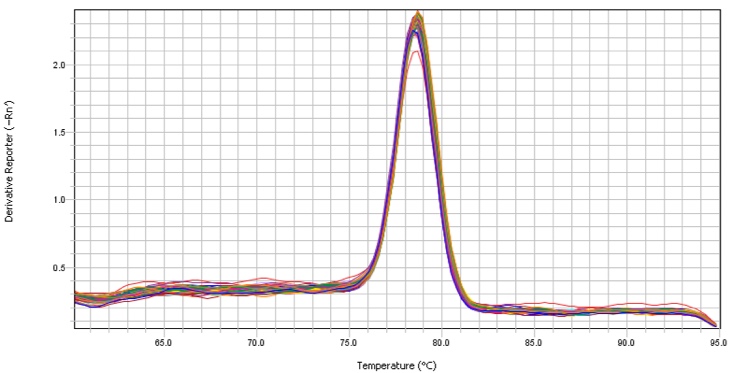


BDNF Variant 5


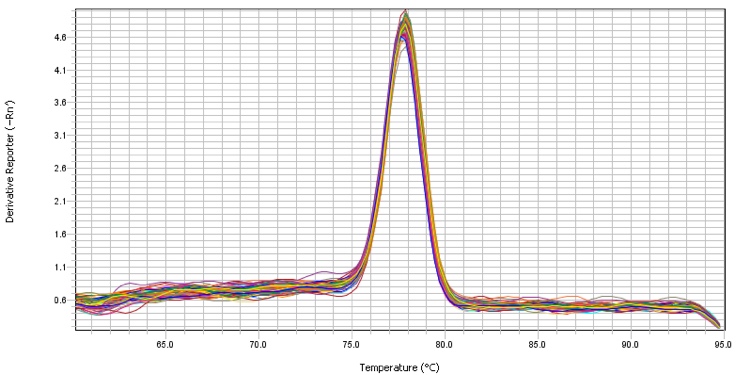


Pan BDNF


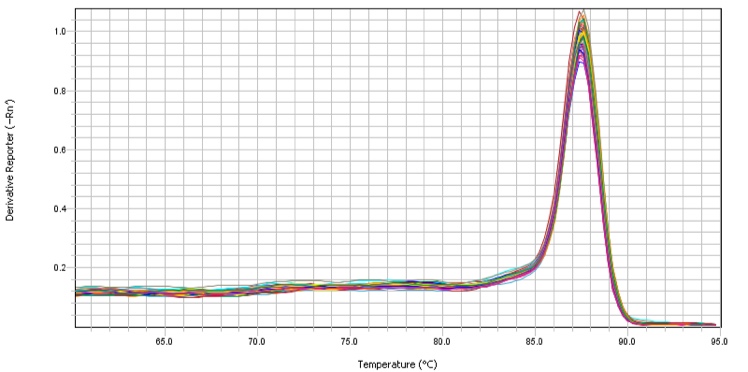


BDNF-AS


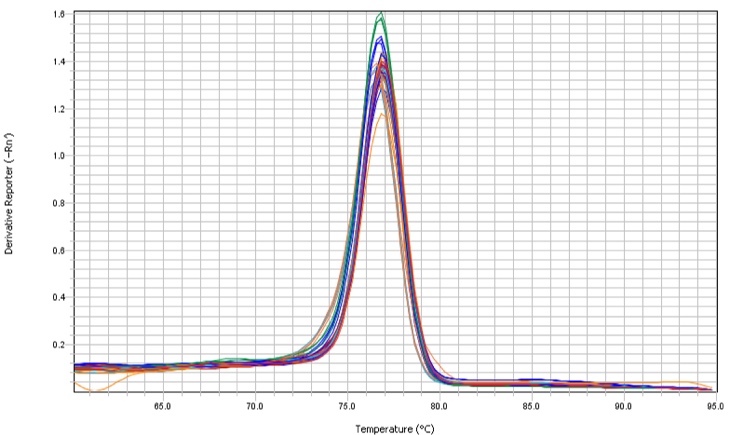


PSMC4


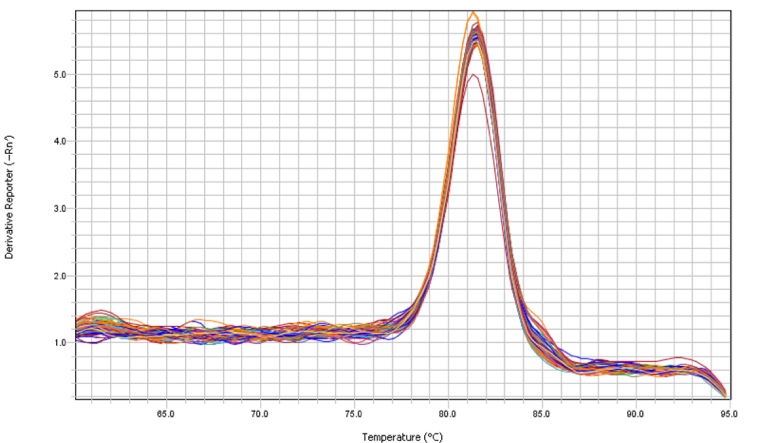


EIF28I


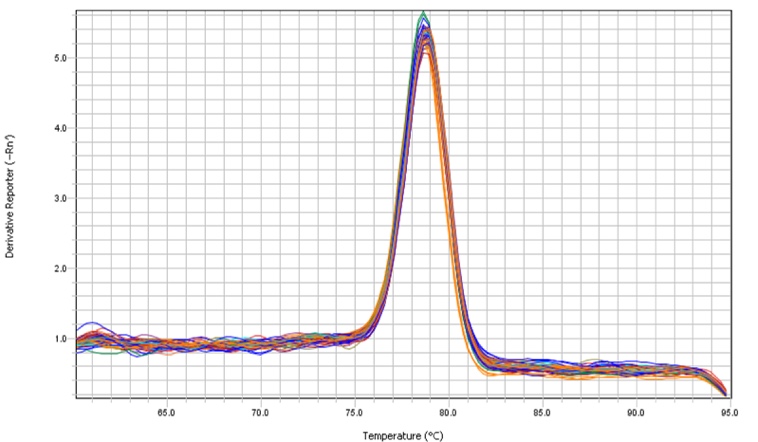


CDNK2B


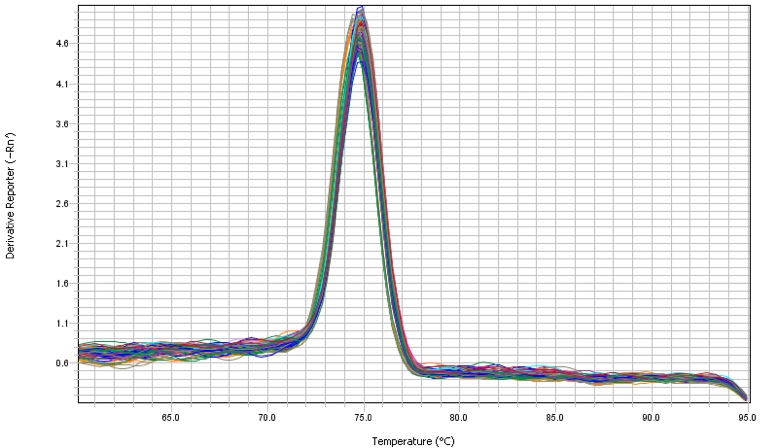


**Figure 2: Melt curve analysis of qPCR of BDNF Variant 4 cDNA revealed two peaks, indicated by red arrows.** Following the analysis, the qPCR products were combined and electrophoresed to determine whether the peaks represent one or more amplicons.


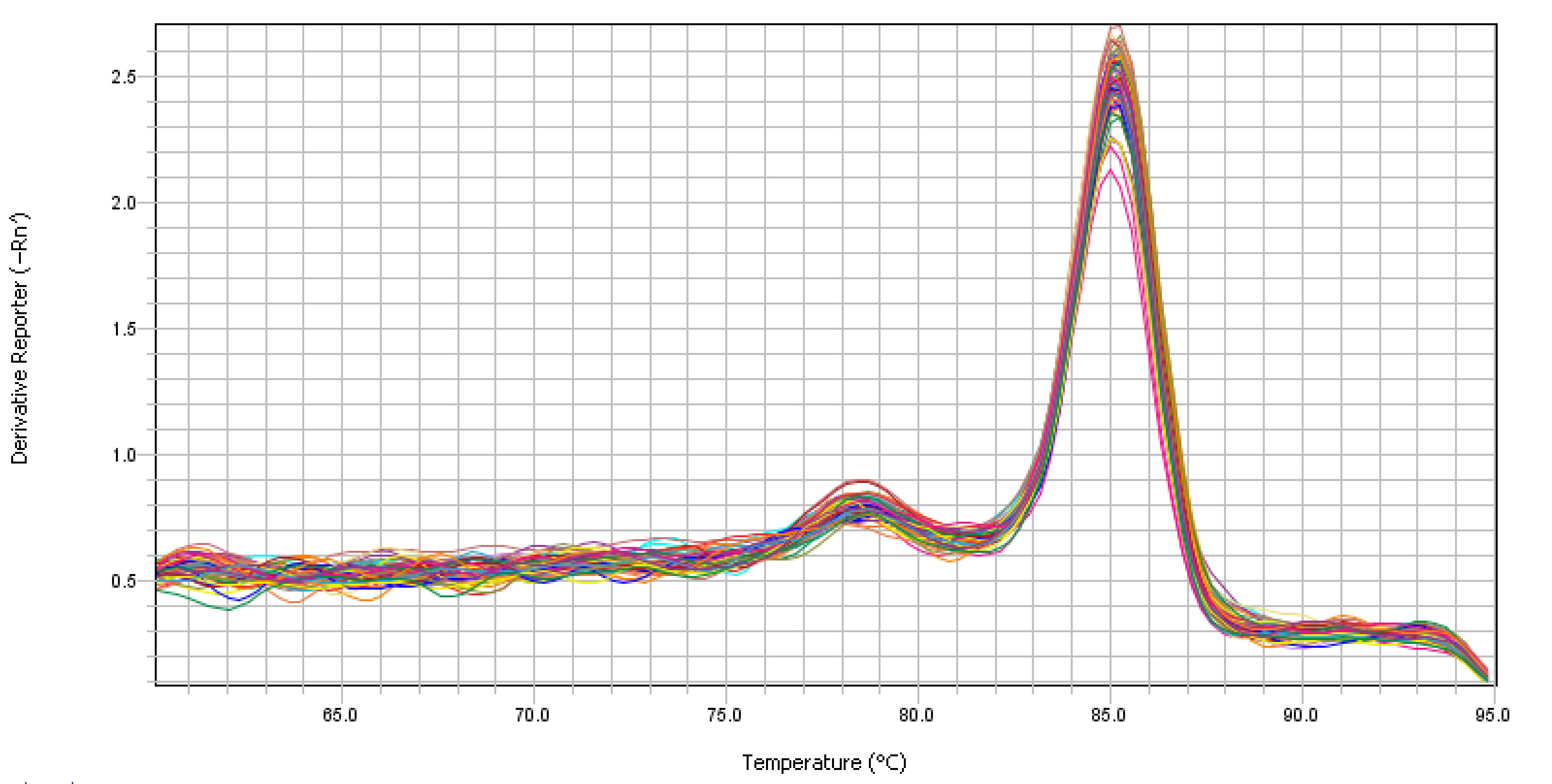

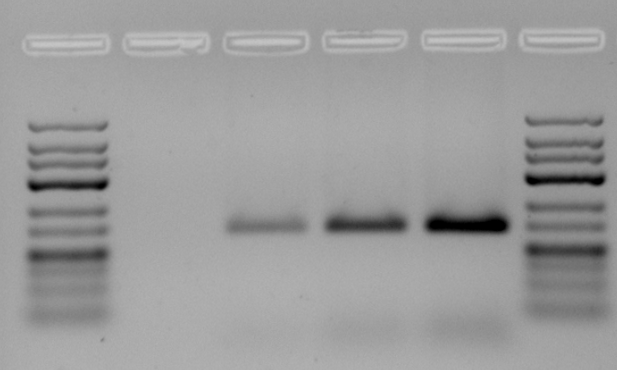


1

2

3

4

5

6

100 bp

150 bp

200 bp

**Agarose gel electrophoresis of qPCR of BDNF Variant 4 cDNA shows a single band representing a single amplicon.** qPCR from BDNF Variant 4 cDNA was electrophoresed on a 2% agarose gel in 1X TBE buffer. A single band appears at ~ 143 bp, the amplicon length for the BDNF Variant 4 Taqman assay (ThermoFisher, #Hs00156058_m1) per the manufacturer.

**Lane 1:** DNA ladder (Thermo Scientific GeneRuler Low Range DNA Ladder,#FERSM1193)

**Lane 2**: no qPCR product

**Lane 3:** 5 μl of qPCR product

**Land 4:** 10 μl of qPCR product

**Lane 5:** 15 μl of qPCR product

**Lane 6:** DNA ladder (Thermo Scientific GeneRuler Low Range DNA Ladder,#FERSM1193)
